# Virtual 3D surface imaging system for the validation of tumor tracking algorithm used in surface‐guided radiotherapy

**DOI:** 10.1002/acm2.70290

**Published:** 2025-11-07

**Authors:** Xiaolong Wu, Ziwen Wei, Shaozhuang Zhai, Yang Zhang, Zhihua Liu, Tao Jiang, Lei Zhang, Junchao Qian

**Affiliations:** ^1^ School of Biomedical Engineering Anhui Medical University Hefei China; ^2^ Anhui Province Key Laboratory of Medical Physics and Technology Institute of Health and Medical Technology Hefei Institutes of Physical Science Hefei Cancer Hospital Chinese Academy of Sciences Hefei P.R. China; ^3^ Information Materials and Intelligent Sensing Laboratory of Anhui Province Anhui University Hefei P.R. China

**Keywords:** phase measurement profilometry, surface imaging, surface‐guided radiotherapy, unity, virtual simulation

## Abstract

**Purpose:**

To develop a surface 3D reconstruction simulation system based on the Phase Measurement Profilometry (PMP) and the Unity physics engine and validate its feasibility for assisting in the development of the Surface‐Guided Radiotherapy (SGRT) tumor tracking algorithm.

**Methods:**

The components, such as cameras and projectors, are set up in the Unity environment to enable structured‐light‐based surface 3D reconstruction simulation using the PMP. This process includes structured light projection, camera calibration, phase unwrapping, and point cloud reconstruction procedures. The influence of parameter settings on the effectiveness of 3D reconstruction is investigated, including different distances and angles between the camera and the measurement surface, as well as variations in light intensity. The simulation capabilities of the system are validated by comparing surface imaging of the same human torso model in a radiotherapy room environment and within the simulation system. Additionally, the simulation system is further utilized to acquire surface imaging data required for the SGRT tumor tracking algorithm. A comparison is made between this data and the idealized skin surface imaging data obtained directly from CT reconstruction segmentation to verify the system's supportive role in the development of the SGRT tumor tracking algorithm.

**Results:**

The effects of varying light intensity and object positioning in the simulation system are consistent with those reported in previous studies conducted in real‐world environments. The root mean square errors (RMSE) of the surface imaging point clouds from different perspectives between the simulation system and the actual environment are 0.46, 0.47, and 0.52 mm, all at sub‐millimeter levels. The validation in the development of the SGRT tumor tracking algorithm indicates that the simulation system enables the SGRT algorithm development to avoid relying on overly idealized surface imaging data.

**Conclusions:**

The simulation system based on PMP and Unity has been proposed, enabling a broader range of measurement conditions to be set in the virtual environment, thereby saving costs for measurements in real‐world scenarios. This system can also be utilized to assist in the validation of the SGRT tumor tracking algorithms, thereby advancing progress in this field.

## 1 INTRODUCTION

Phase Measurement Profilometry (PMP) is a widely used structured light 3D reconstruction technique for 3D shape measurement, which utilizes a structured light projector to yield specific encoded patterns and calculates the phase to obtain 3D shape information of the surface of the object.^1^ PMP plays a significant role in various fields such as computer vision, medical surgery, intelligent manufacturing, and industrial applications.^2^, ^3^, ^4^, ^5^, ^6^, ^7^ Additionally, the structured light system has found new applications in biomedical fields, such as in surgical navigation,^8^ 3D measurement of otoscopes,^9^ and 3D multimodal medical imaging.^10^

The Surface‐Guided Radiotherapy (SGRT) algorithm refers to the tracking of patient surface motion and positioning during radiotherapy using surface 3D imaging data obtained through 3D surface reconstruction algorithms. This ensures precise radiation delivery to tumor areas.^11^ And the 3D surface reconstruction algorithm is the method used to acquire surface 3D imaging data. In the research related to SGRT algorithms, surface imaging plays a crucial role, with the raw data typically being highly idealized point cloud data. For example, Huang et al.^12^ utilized the registration of overly idealized point clouds segmented from CT thresholding to generate a displacement field for synthesizing real‐time CT images. Wei et al.^13^ estimated real‐time CT using highly idealized skin surfaces reconstructed from planned 4DCT. Zhai et al.^11^ Utilized surface imaging point clouds obtained by actual scanning, employing iterative closest point (ICP) registration and augmented reality to assist with radiotherapy positioning. Although the point cloud data is not idealized, the process of surface imaging experimentation is rather cumbersome. Therefore, this work aims to present an efficient and cost‐effective verification tool for the development of SGRT algorithms. This tool enables the development of SGRT algorithms without the need to set up an actual surface imaging system, and avoids the use of overly idealized raw data (such as surface imaging directly segmented from CT reconstructions). Given that surface imaging systems are inevitably used in clinical applications, the primary goal of the simulation system is to reduce ineffective iterations by filtering out flawed algorithms prior to clinical trials, thus contributing to the advancement of SGRT algorithms. The system can support various types of SGRT algorithms, including tracking lung tumors during respiratory motion, patient initial setup monitoring, and real‐time chest CT estimation. The surface imaging simulation system proposed in this paper has been validated for the SGRT tumor tracking algorithm. Different 3D imaging methods have been proposed, among which time‐of‐flight,^14^ stereoscopic vision,^15^ and near infrared light^16^ are widely applied. Time‐of‐flight technology requires precise specifications for its timing measurement module. Despite its capability to swiftly capture 3D data, the resolution remains relatively low.^4^ Stereoscopic vision matches images based on visual features. Lack of features can lead to matching failures, high computational complexity, and large computational requirements,^4^ making it unsuitable for scenes with monotonous or texture‐deficient characteristics.^17^ Structured light PMP acquires detailed surface information by analyzing phase differences, providing superior capturing capabilities for subtle surface features. It offers the advantages of high resolution and precision.^18^ Structured light surface imaging is a non‐ionizing radiation, non‐invasive technique that can be employed for continuous patient position monitoring during radiotherapy procedures in different body regions, including the chest, head and neck, and limbs.^19^ It offers advantages such as high accuracy, strong robustness, simplicity, and non‐contact operation.^3^, ^20^

However, traditional structured light sensors require operation in relatively controlled lighting environments and are susceptible to complex lighting interferences, which makes it challenging to obtain optimal measurement results.^21^ The accuracy of reconstruction is influenced by various factors, including the projected light source, camera resolution, and object positioning characteristics. Consequently, the optimal setup for a structured light phase measurement system varies depending on the specific circumstances, necessitating effective measurement validation to guarantee the accuracy and reliability of the results. However, experimental validation using a trial‐and‐error approach for practical measurement systems can be time‐consuming and costly. Thus, it is necessary to utilize computer simulations for easier verification of the system.^22^

Unity provides a comprehensive graphical user interface (GUI) and advanced computer graphics processing capabilities, allowing for the creation of virtual 3D environments as a game engine.^23^, ^24^, ^25^ It is capable of simulating 3D measurement spaces that closely resemble real scenarios, including the emulation of cameras, projectors, light sources, objects, and capturing operations. Unity is equipped with an advanced high‐definition rendering pipeline (HDRP) and custom shader programming. It boasts a rich array of physical materials and lighting models, enabling accurate simulation of complex imaging in various environments. Currently, Unity is being utilized in simulations for target detection,^26^ interactive driving,^27^ mechanical design,^28^ internal medicine surgery,^29^ and other fields.

We conduct 3D reconstruction using the camera‐projector triangulation model, where camera‐projector calibration is a fundamental and crucial step in structured light 3D measurement.^30^ The calibration process involves collecting a set of images to complete.^31^ This set of images comprises 20 calibration board images of a circular pattern captured at various distances and angles. The images exhibit uniform lighting, high resolution, clear and evenly distributed visible feature points, and distinct geometric structures, all intended to enhance the precision and stability of the imaging system. We employ a phase measurement method to accomplish structured light‐based surface 3D reconstruction within the Unity spatial environment. To validate our approach, we conduct measurements on various objects with different shapes and sizes, as well as a human torso model. Through simulation experiments, we replicate processes such as structured light projection, phase decoding, and camera calibration under diverse conditions. We investigated the impact of parameter settings, including different distances and angles between the camera and the measured surface, as well as variations in light source intensity, on the effectiveness of 3D reconstruction. We employ effective filtering and gray code methods during data processing to enhance the accuracy and robustness of the measurement results, providing theoretical guidance for practical system design. In addition, we compared the surface imaging of the same human torso model in a radiotherapy room environment and the simulation system to demonstrate the modeling capability of the simulation system. Furthermore, we utilized the simulation system as an optical imaging device to conduct tumor estimation processes during the development of SGRT tumor tracking algorithms, acquiring 3D images of the skin surface. This validation was conducted to confirm the supportive role of the system in the development of SGRT algorithms.

## 2 UNITY SIMULATION

### 2.1 Simulation space

**FIGURE 1:**
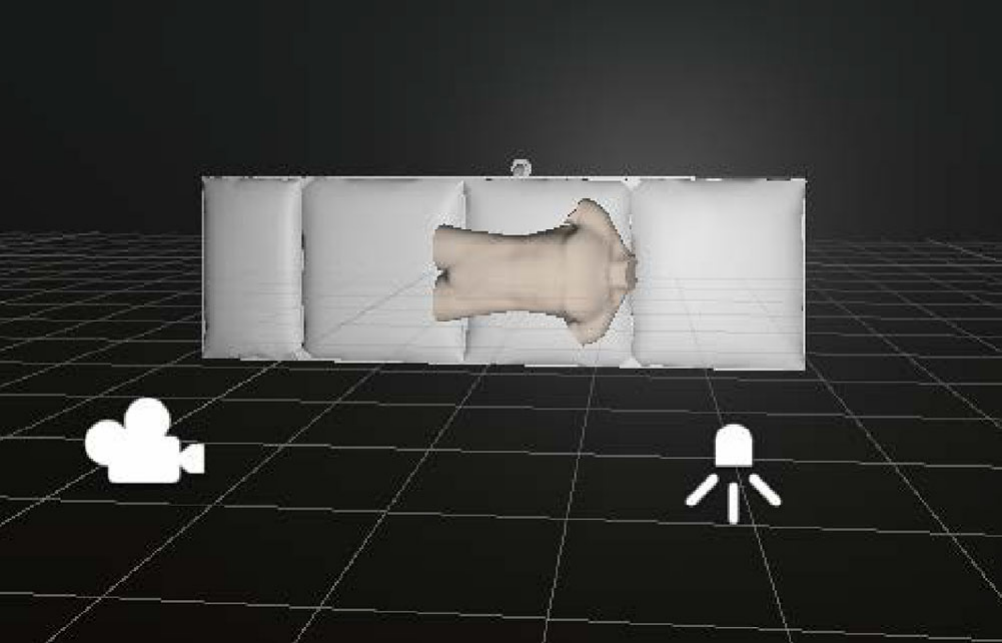
Measurement system built using the simulator. The system comprises a camera, a projector, and a constructed simulation model.

Within a unified simulation space, the positioning of 3D CAD models and light sources can be freely accomplished. Moreover, setting the key parameters of the camera and projector in Unity, such as pixel size, grating signal, throw ratio, light intensity, focal length, exposure time, distortion, depth‐of‐field, noise intensity, image sensor size, specular reflection, etc., can simulate the main characteristics of the camera and projector in the PMP measurement system. The measurement system used to simulate a radiotherapy room is illustrated in Figure 1. Unity further enables GUI operations and provides control over the physics engine, offering ease of use and flexibility in manipulating the simulated environment.

### 2.2 System simulation process

**FIGURE 2:**
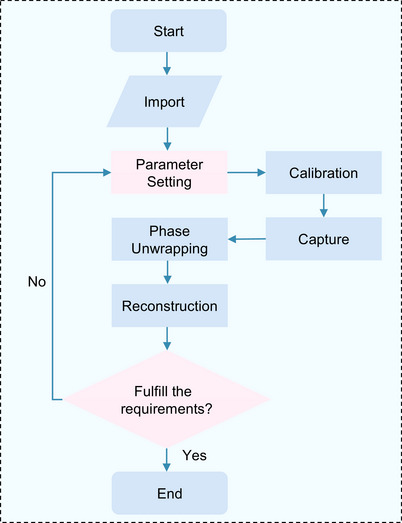
Flowchart of the measurement process in the proposed system. The flowchart encompasses model importing, parameter setting, system calibration, image capture, phase unwrapping, and reconstruction measurement processes.

The process involves five steps based on Unity components and the measurement procedure. Figure 2 depicts the flowchart of the proposed simulator. Step 1: Utilize the 3D modeling software Blender^32^ to create a CAD model or obtain one from a 3D model library. Import the models into Unity and position them within the simulated space. Step 2: Adjust the measurement parameters. Step 3: Project stripes onto the object using the projector and capture images of the object using the camera. Step 4: Compute the 3D shape of the object through phase unwrapping and system calibration techniques. Step 5: Analyze the measurement results and fine‐tune the parameters to enhance accuracy. Upon achieving the desired outcomes, finalize the system configuration.

## 3 3D RECONSTRUCTION SIMULATION

### 3.1 Structured light 3D reconstruction algorithm

**FIGURE 3:**
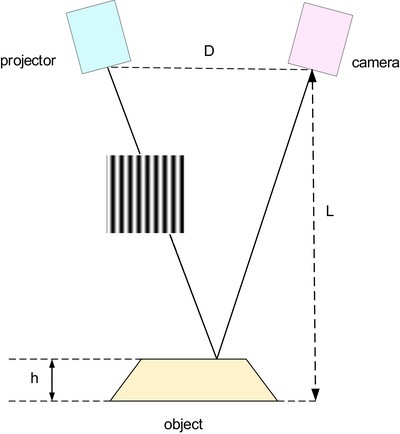
Schematic of the PMP. In the description of the 3D reconstruction measurement, the variables D and L represent the distances between the camera and the projector, and between the camera and the measurement surface, respectively. The parameter *h*(*x*, *y*) denotes the height of the object. By employing a structured light projector to generate specific encoding patterns and calculating phases, it can obtain 3D shape information of the object's surface. PMP, Phase Measurement Profilometry.

Figure 3 depicts the configuration of the reconstruction system. The variables D and L represent the distances between the camera and the projector, as well as between the camera and the measurement surface, respectively. The parameter *h* (x, y) represents the height of the object. Furthermore, a pinhole model is employed in the proposed simulator. The imaging principle involves the formation of an inverted and reduced image as light passes through a very small pinhole. The fringe patterns with sinusoidal intensity changes are projected onto the measurement object within the reconstruction system. When captured by the camera positioned away from the camera's optical axis, distorted stripes appear on the object's surface. The captured images can be formulated as follows:

(1) $$I\left(x,y\right)=A\left(x,y\right)+B\left(x,y\right)\cos(2\pi f_{0}x+\varphi \left(x,y\right))$$

The variables (*x*, *y*) represent the coordinates of the image. The parameters A (*x*, *y*), B (*x*, *y*), *f*_0_, and *φ* (*x*, *y*) correspond to the DC component, which is the background light intensity, stripe amplitude, spatial frequency, and phase variation of the object's surface, respectively. By applying image processing techniques to determine the phase *φ* (*x*, *y*) for each pixel, the shape of the object can be measured. The *N*‐step phase‐shifting profilometry (*N*‐step PSP) is a high‐precision phase calculation method that utilizes *N* images, where the stripe pattern is shifted by 2π/*N*. The mathematical representation of the *n*‐th image can be expressed as:

(2) $$I_{n}\left(x,y\right)=A\left(x,y\right)+B\left(x,y\right)\cos\left(2\pi f_{0}x+\varphi \left(x,y\right)+\frac{2\pi n}{N}\right)$$

Assuming that the DC component, A (*x*, *y*), and the amplitude, B (*x*, *y*), remain constant across *N* images, the phase *φ* (*x*, *y*) can be calculated using the following equation:

(3) $$\varphi \left(x,y\right)=\arctan\frac{\sum_{n=0}^{N-1}I_{n}\left(x,y\right)\sin\left(\frac{2\pi n}{N}\right)}{\sum_{n=0}^{N-1}I_{n}\left(x,y\right)\cos\left(\frac{2\pi n}{N}\right)}$$

The phase values obtained are wrapped within the range of (−π/2, π/2) due to the arctan function. Consequently, phase unwrapping is necessary to convert the wrapped phase values into their actual values.^33^ Following the phase unwrapping process, *h* (*x*, *y*) is determined based on the geometric conditions of the reconstruction system. The 3D shape of the object is subsequently calculated using the following formula:

(4) $$h\left(x,y\right)=\frac{L\varphi (x,y)}{2\pi f_{0}D+\varphi \left(x,y\right)}$$

During the phase unwrapping process, gray codes are employed as an auxiliary tool to facilitate hierarchical unwrapping. The formula is as follows:

(5) $$U\left(x,y\right)=\sum_{i}^{j}GC_{i}\left(x,y\right)*2^{\left(j-i\right)}$$

(6) k(x,y)=i(U(x,y))

(7) $$\Phi^{s}\left(x,y\right)=\varphi \left(x,y\right)+2\pi k\left(x,y\right)$$

The m‐bit gray code patterns are utilized to label phase levels, denoted by *k*, with truncation up to 2*^j^*. The variable *i*(.) is utilized to determine the established relationship between the encoded decimal number *U* (x, y) and the phase order *k* (x, y). The *k*_1_ and *k*_2_ represent the first and second levels of the Gray code, respectively. Formula (7) is employed to unwrap the truncated phase.

(8) $$\Phi^{s}\left(x,y\right)=\left\{\begin{array}{c} \varphi \left(x,y\right)+2\pi k_{2}\left(x,y\right),\;\varphi \left(x,y\right)⩽-\frac{\pi }{2}\\ \varphi \left(x,y\right)+2\pi k_{1}\left(x,y\right),\;-\frac{\pi }{2}<\varphi \left(x,y\right)<\frac{\pi }{2}\\ \varphi \left(x,y\right)+2\pi k_{2}\left(x,y\right)-2\pi ,\;\varphi \left(x,y\right)⩾\frac{\pi }{2} \end{array}\right.$$

Taking a sinusoidal fringe pattern with 16 cycles as an example, four sets of gray codes are required to label the fringe levels and assist in truncating the phase for phase unwrapping.^34^ Once the final unwrapped phase is obtained, the object's 3D shape information can be calculated using phase height mapping techniques and camera calibration procedures.

System Calibration: Mapping the world coordinate system [*X_w_, Y_w_, Z_w_*]*^T^* of the object to the coordinates [*u, v*]*^T^* in the pixel coordinate system:

(9) $$Z_{c}\left[\begin{array}{c} u\\ v\\ 1 \end{array}\right]=\left[\begin{array}{cccc} a_{11}^{c}, & a_{12}^{c}, & a_{13}^{c}, & a_{14}^{c}\\ a_{21}^{c}, & a_{22}^{c}, & a_{23}^{c}, & a_{24}^{c}\\ a_{31}^{c}, & a_{32}^{c}, & a_{33}^{c}, & a_{34}^{c} \end{array}\right]\left[\begin{array}{c} X_{w}\\ Y_{w}\\ Z_{w}\\ 1 \end{array}\right]$$

There is a relationship between the world coordinate system and the pixel coordinate system of the camera projector setup as follows: camera coordinates [*u^c^*, *v^c^*] and projector coordinates [*u^p^*, *v^p^*];

(10) $$\begin{array}{c} s^{c}{\left[u^{c},v^{c},1\right]}^{T}=A^{c}{\left[X_{W},Y_{W},Z_{W},1\right]}^{T}\\ s^{p}{\left[u^{p},v^{p},1\right]}^{T}=A^{p}{\left[X_{W},Y_{W},Z_{W},1\right]}^{T} \end{array}$$

For a given phase value $\phi_{a}$, the following relationship holds:

(11) $$\phi_{a}\left(u^{c},v^{c}\right)=\phi_{a}^{p}\left(u^{p}\right)=\phi_{a}$$

The projected fringe pattern consists of uniform stripes, where W represents the field of view width, *ϕ* represents the absolute phase, and *ϕ*/2π represents the phase ratio. Therefore, we have the following relationship:

(12) $$u^{p}=\frac{\phi_{a}^{p}\left(u^{p}\right)}{2\pi }W=\frac{\phi_{a}^{c}\left(u^{c},v^{c}\right)}{2\pi }W$$

By combining the previous Equations (9)–(12), we obtain:

(13) $$\begin{array}{cc} \left[\begin{array}{c} X_{W}\\ Y_{W}\\ Z_{W} \end{array}\right] & ={\left[\begin{array}{ccc} a_{11}^{c}-u^{c}a_{31}^{c} & a_{12}^{c}-u^{c}a_{32}^{c} & a_{13}^{c}-u^{c}a_{33}^{c}\\ a_{21}^{c}-v^{c}a_{31}^{c} & a_{22}^{c}-v^{c}a_{32}^{c} & a_{23}^{c}-v^{c}a_{33}^{c}\\ a_{11}^{p}-u^{p}a_{31}^{p} & a_{12}^{p}-u^{p}a_{32}^{p} & a_{13}^{p}-u^{p}a_{33}^{p} \end{array}\right]}^{-1}\\ & \times \left[\begin{array}{c} u^{c}a_{34}^{c}-a_{14}^{c}\\ v^{c}a_{34}^{c}-a_{24}^{c}\\ u^{p}a_{34}^{p}-a_{14}^{p} \end{array}\right] \end{array}$$

**FIGURE 4:**
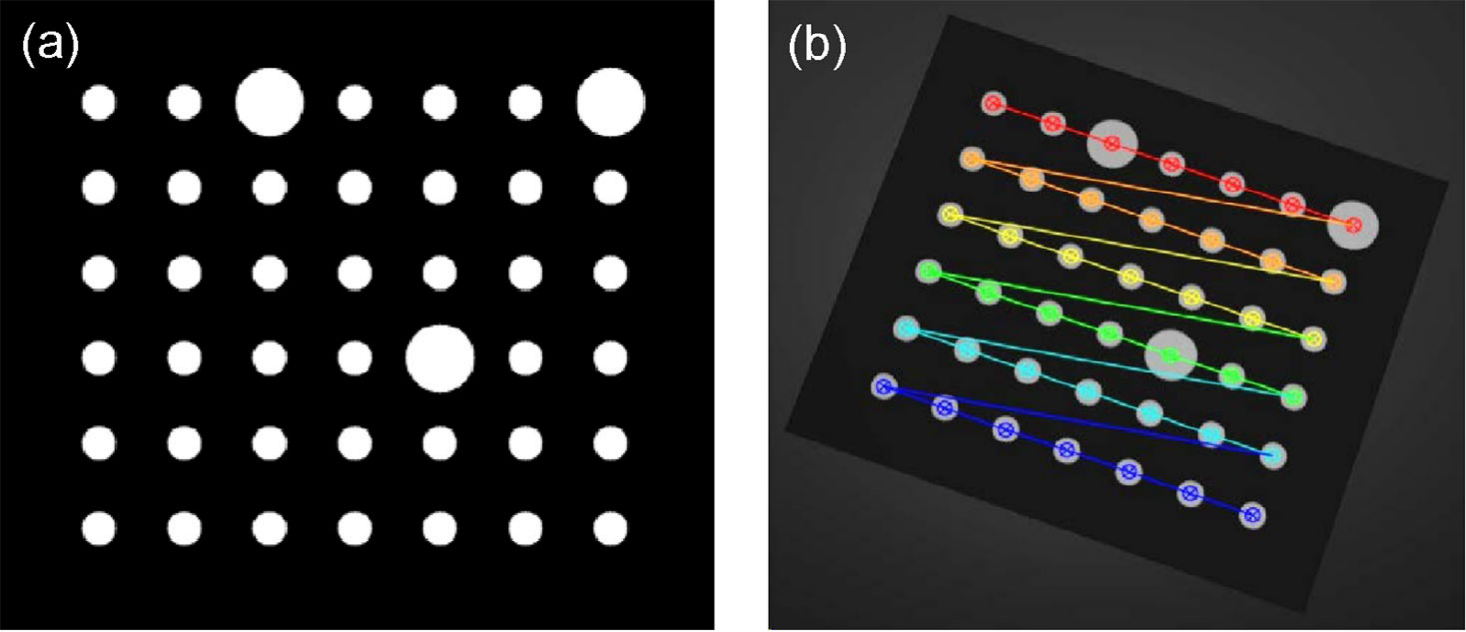
(a) Calibration Board, (b) Corner Extraction. By capturing the dot patterns on the calibration board, the world coordinates and pixel coordinates of the dot centers are obtained under the camera and projector, enabling the calibration of intrinsic and extrinsic parameters.

**FIGURE 5:**
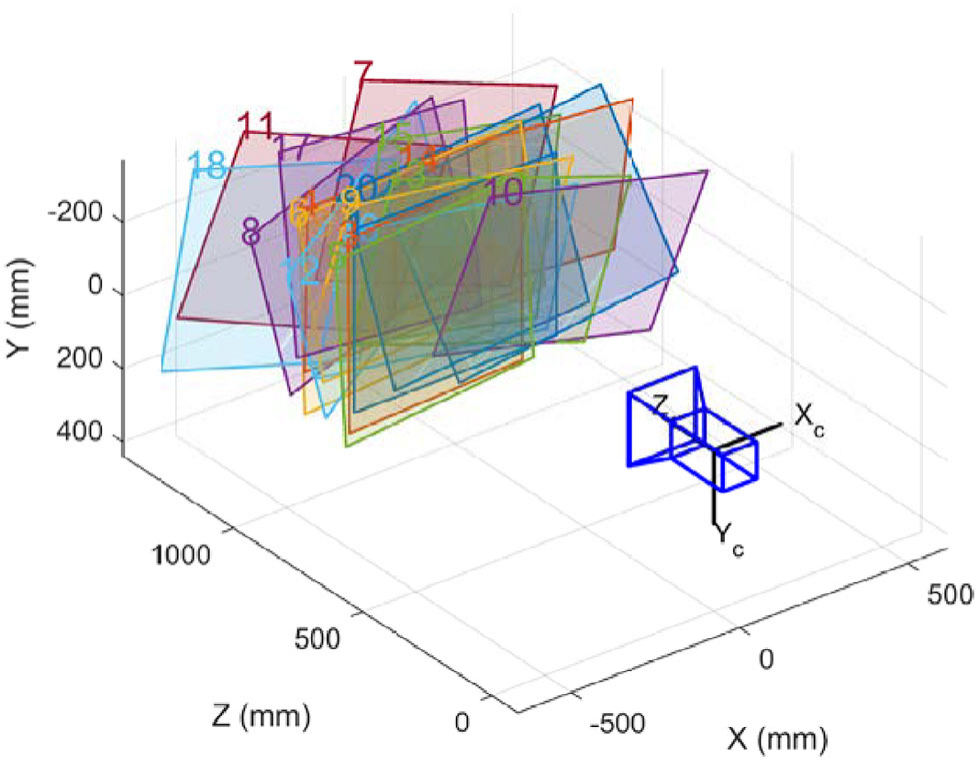
Camera calibration external parameters. The parameters that describe the position and orientation of the camera in the world coordinate system determine the viewpoint and position from which the camera observes the world.

**FIGURE 6:**
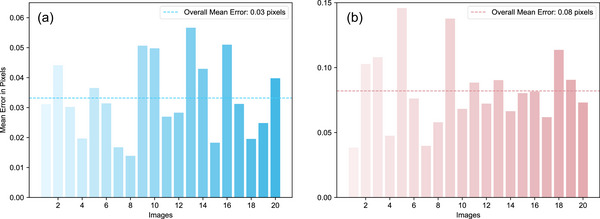
(a) Reprojection error of camera, (b) Reprojection error of projector. The reprojection error is a crucial indicator used in camera calibration to assess calibration accuracy. The overall average reprojection errors for the camera and projector are 0.03 pixels and 0.08 pixels, respectively.

To attain a more precise reconstruction of the 3D point cloud, the system calibration^35^ was executed. Figure 4 illustrates a schematic diagram of the calibration board and the extracted corner points before calibration. The large and small circular dots, with diameters of 5 and 2.5 mm, respectively, are clearly visible at specific distances and under certain environmental conditions. The larger dots are utilized for scale calibration and positioning markings. By capturing the dot patterns on the calibration board, the world coordinates and pixel coordinates of the dot centers are obtained under the camera and projector, enabling the calibration of intrinsic and extrinsic parameters. Figure 5 shows the extrinsic calibration of the camera, while the calibration errors of the camera and the projector are shown in Figure 6. The overall average reprojection error for the camera and the projector is 0.03 pixels and 0.08 pixels, respectively. The measurement setup is arranged in such a manner that enables the camera to capture the entire object within its field of view. Specifically, the camera focal length and optimal field of view may vary depending on the measurement object and lighting conditions.^18^

### 3.2 Parameter settings and measurement in the simulation system

**FIGURE 7:**
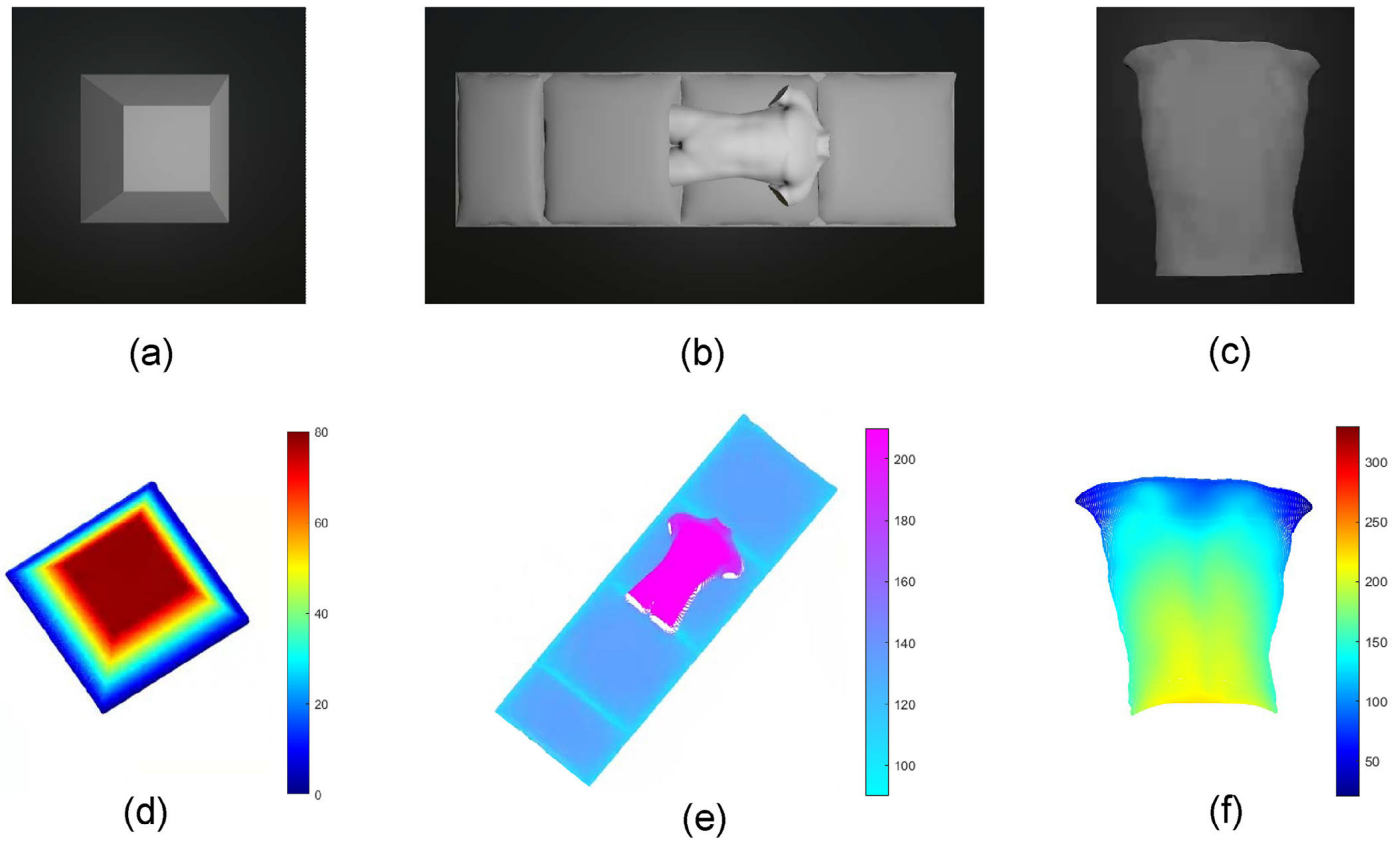
Model image from camera perspective: (a) Trapezoid, (b) Treatment bed, and (c) Torso; Reconstruction results: (d) Trapezoid, (e) Treatment bed, and (f) Torso.

The proposed simulation system was utilized to validate the measurement setup. In this study, a 12‐step PSP (*N *= 12) and gray codes were employed. The 3D CAD object underwent precise geometric measurements, and the measurement results were refined based on different parameter configurations to further optimize the simulation system. During the simulation, the camera lens was set to a focal length of 20 mm. The camera sensor size is 36 mm × 24 mm, capturing images at a resolution of 1138 pixels × 489 pixels. The projector had a pixel number of 1920 pixels × 1080 pixels, and its aspect ratio was 8:12. The device was initially positioned at D = 400 and L = 1000 mm. The fringe frequency was configured as *f*_0 _= 0.172 lp/mm, reflecting the periodic structural variations in the fringe patterns. Figure 7 shows the measurement results, which include the images captured by the camera in the virtual 3D space and the reconstructed image.

## 4 RESULTS AND ANALYSIS OF THE SIMULATION EXPERIMENTS

In this section, multiple simulations have been discussed. Section 4.1 discusses the impact of different parameters on the measurement results. Section 4.2 quantitatively demonstrates how the simulation system well mimics an actual system. Section 4.3 validates the applicability of the simulation system in the development of SGRT algorithms.

### 4.1 The influence of different parameters

#### 4.1.1 The adjustment of relative position

**FIGURE 8:**
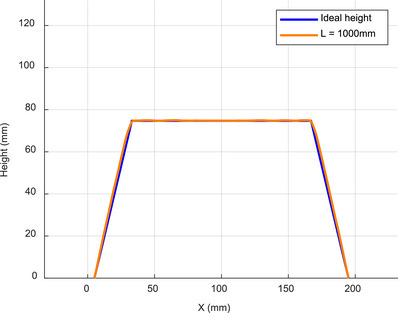
Cross‐sectional view of the Trapezoid. Comparison of the height measurement results at *L* = 1000 mm with the ideal height.

**FIGURE 9:**
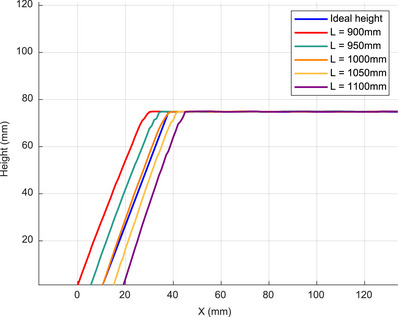
Comparison of measurement results at different positions of the object and camera. It shows a magnified view of the corner region, comparing the height measurement results for different *L* values.

**FIGURE 10:**
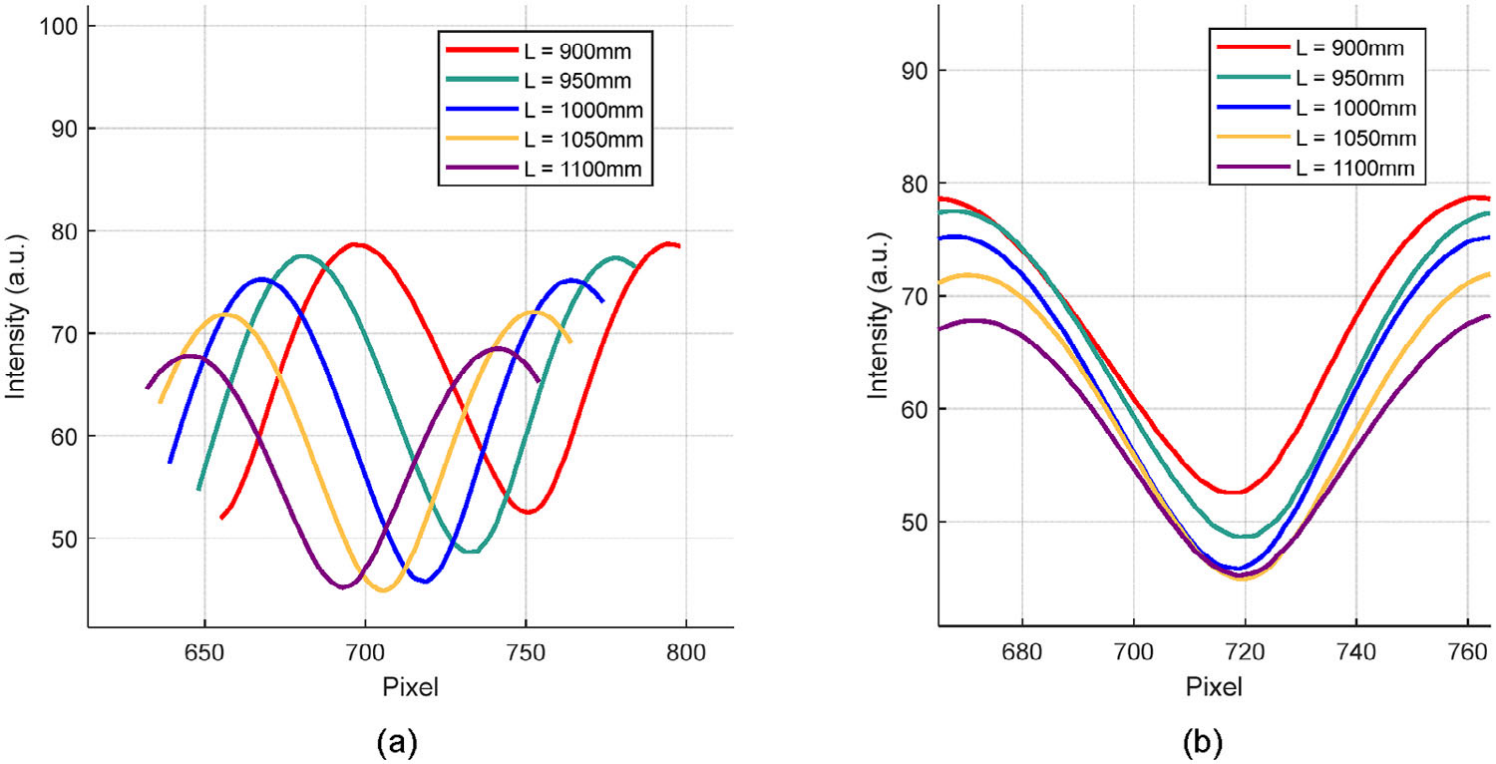
(a) Fringe waveform of stripes captured at different positions, (b) Fringe waveform for stripe at the same pixel. It describes the intensity variations within pixel values of modulated patterns at different distances before phase unwrapping.

In the simulation of a regular trapezoid, the camera and projector were positioned at *L* = 1000 and *D* = 400 mm, respectively, while the remaining settings remained the same. Figure 8 shows a comparison between the height measurement results at *L* = 1000 mm and the ideal height, which represents the actual height. Figure 9 shows a magnified view of the corner region comparing the height measurement results for different L values. Figure 10 describes the intensity variations within pixel values of modulated patterns at different distances before phase unwrapping. Due to the steeper intensity variation curve, it can be observed that the error is smaller and closer to the original object model when *L* = 1000 mm. The mean absolute error (MAE), Hausdorff distance (HD), and mean Distance to Agreement (MDTA) are used to evaluate the accuracy of the PMP system. The maximum and average minimum distances from a point in one set to any point in another set are represented by HD and MDTA, respectively. They are utilized for comparing the similarity or consistency between two sets. The cross‐sectional mean absolute error (CMAE) represents the average absolute distance between the reconstructed trapezoid cross‐sectional curve and the original model's cross‐sectional curve. The MAE represents the average absolute distance between the reconstructed trapezoidal and the original model. The comparison of the measurement results of the trapezoid at different distances with the original model is shown in Table 1. Compared to other positions, the error is minimal at *L* = 1000 mm. This finding is consistent with the previous study,^36^ which reported that both far and close distances in the actual environment have an impact on data quality.

**TABLE 1:** Comparison of measurement results of the trapezoids at different distances with the original model.

Position (100 mm)	CMAE (mm)	MAE (mm)	HD (mm)	MDTA (mm)
**9**	7.16	7.24	10.28	5.86
**9.5**	3.48	3.73	5.36	3.61
**10**	0.52	0.68	1.63	0.59
**10.5**	3.29	3.67	5.24	3.35
**11**	7.31	7.36	11.03	6.22

Abbreviations: CMAE, cross‐sectional mean absolute error; HD, Hausdorff distance; MAE, mean absolute error; MDTA, mean Distance to Agreement.

**FIGURE 11:**
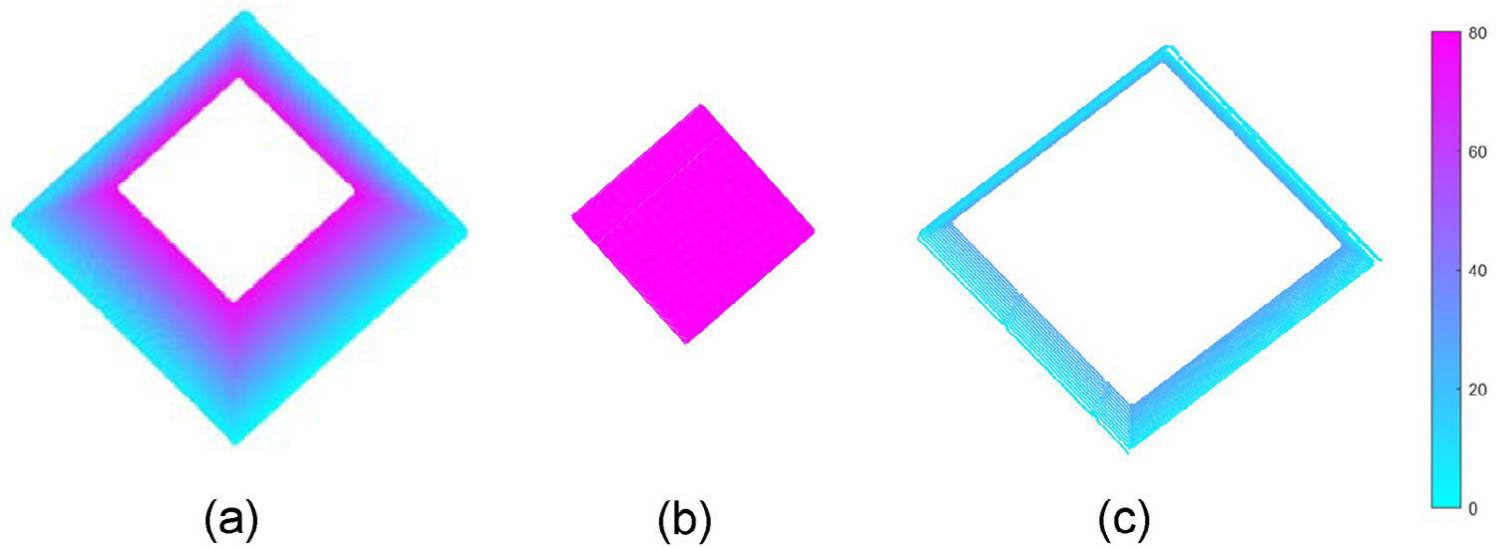
The reconstructed segmentation diagram of the trapezoid. It illustrates the distinct structures of the reconstructed trapezoid when the distance L from the camera to the measurement surface is 1000 mm. Slice the trapezoid along the horizontal plane at *z* = 70 mm to obtain the edge perimeter part (a) and the center part (b), then slice along the horizontal plane at *z* = 20 mm, to obtain the bottom edge part (c).

To explore the differences in measurement precision among the distinct structures of the regular trapezoid, measurement precision values for the different parts of the trapezoid were calculated at *L* = 1000 mm. By cutting along the horizontal plane at *Z* = 70 mm, we obtained the edge circumference part (as shown in Figure 11a) and the center part (as shown in Figure 11b). Subsequently, cutting along the horizontal plane at *Z* = 20 mm allowed us to obtain the bottom edge part (as shown in Figure 11c). The MAE values corresponding to the measurement results of these three parts were calculated to be 0.70, 0.62, and 0.85 mm, respectively. As illustrated in Figure 11, it is evident that the measurement error of the trapezoid edges is greater than that of the center section, with the measurement error being even more pronounced for the slightly more distant bottom edge.

#### 4.1.2 Perspective factor

**FIGURE 12:**
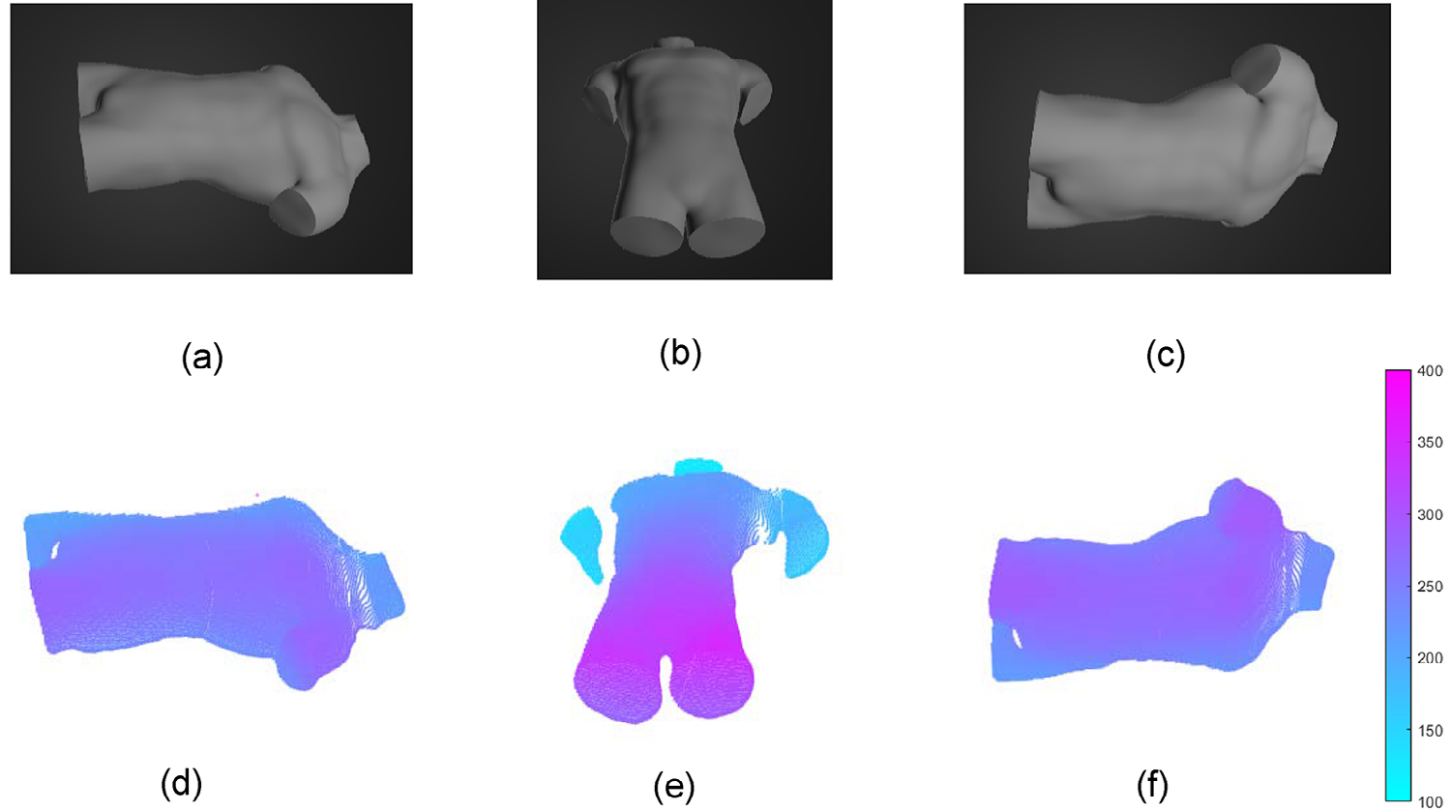
Human torso model image from camera perspective at different angles: (a) The horizontal offset of 45 degrees, (b) The perspective from the foot, (c) The horizontal offset of 135 degrees; Reconstruction results: The (d), (e), and (f) correspond to (a), (b), and (c), respectively.

In addition, in many radiotherapy settings, the treatment head of the LINAC may obstruct the camera for a significant amount of the treatment time. We conducted 3D measurements from multiple angles, including the left and right sides of the human torso model and from the foot direction, which better reflect the actual conditions during radiotherapy. It is evident from Figure 12 and Table 2 that the PMP system can still effectively carry out the reconstruction measurements. In Table 2, the labels Left, Right, and Distal correspond to a, b, and c in Figure 12, respectively.

**TABLE 2:** Comparison of measurement results of the human torso model under different measurement angles with the original model.

Angles	MAE (mm)	HD (mm)	MDTA (mm)
**Left**	0.91	2.50	0.74
**Right**	0.85	2.43	0.68
**Distal**	2.05	3.92	1.57

Abbreviations: HD, Hausdorff distance; MAE, mean absolute error; MDTA, mean Distance to Agreement.

#### 4.1.3 Light source intensity adjustment

**FIGURE 13:**
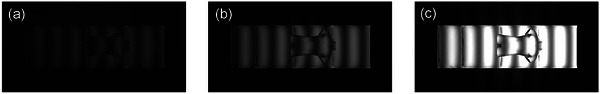
Images taken with different light intensities; (a) Weak light: light intensity value is 0.5, (b) Normal light: light intensity value is 1, (c) Strong light: light intensity value is 2.

The simulator can be configured with various conditions, such as different light source intensities, lens distortions, and camera noise. In this experiment, several light source intensities were set to elucidate the relationship between image intensity and measurement accuracy. The light source intensity was set to 0.5 (weak light), 1 (normal light), and 2 (strong light). Figure 13 displays the images of the virtual human torso model surface modulated by the grating fringe captured under the three different light intensities.

**FIGURE 14:**
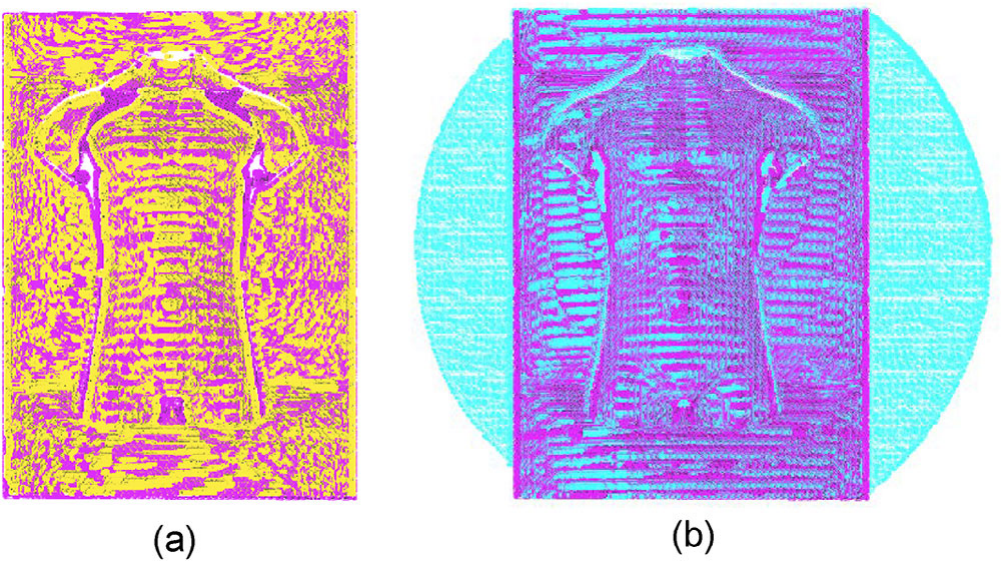
(a) Visual comparison of point cloud reconstruction under normal light (purple) and weak light (yellow) conditions, (b) Visual comparison of point cloud reconstruction under normal light (purple) and strong light (cyan) conditions.

Figure 14 illustrates a simulated comparison of the reconstructed 3D point clouds under three conditions, as shown in Figure 13. According to Table 3, the HD values for the three light intensities are 1.97, 3.06, and 8.53 mm, respectively. It was observed that under the light source intensity condition of 1 (normal light intensity), the completion of surface reconstruction was higher compared to the condition of 0.5 (low light intensity). Additionally, under the light source intensity condition of 2 (high light intensity), the 3D reconstruction resulted in excessive point clouds caused by halo effects, thereby increasing the reconstruction error. This result aligns with the findings of another prior study,^37^ which reported that excessively high or low light intensity in real‐world settings decreases measurement accuracy.

**TABLE 3:** Comparison of measurement results of the human torso model under different lighting conditions with the original model.

Light	MAE (mm)	HD (mm)	MDTA (mm)
**Normal**	0.80	1.97	0.65
**Low**	1.78	3.06	1.64
**High**	5.31	8.53	4.26

Abbreviations: HD, Hausdorff distance; MAE, mean absolute error; MDTA, mean Distance to Agreement.

### 4.2 The degree of simulation of the simulation system

**FIGURE 15:**
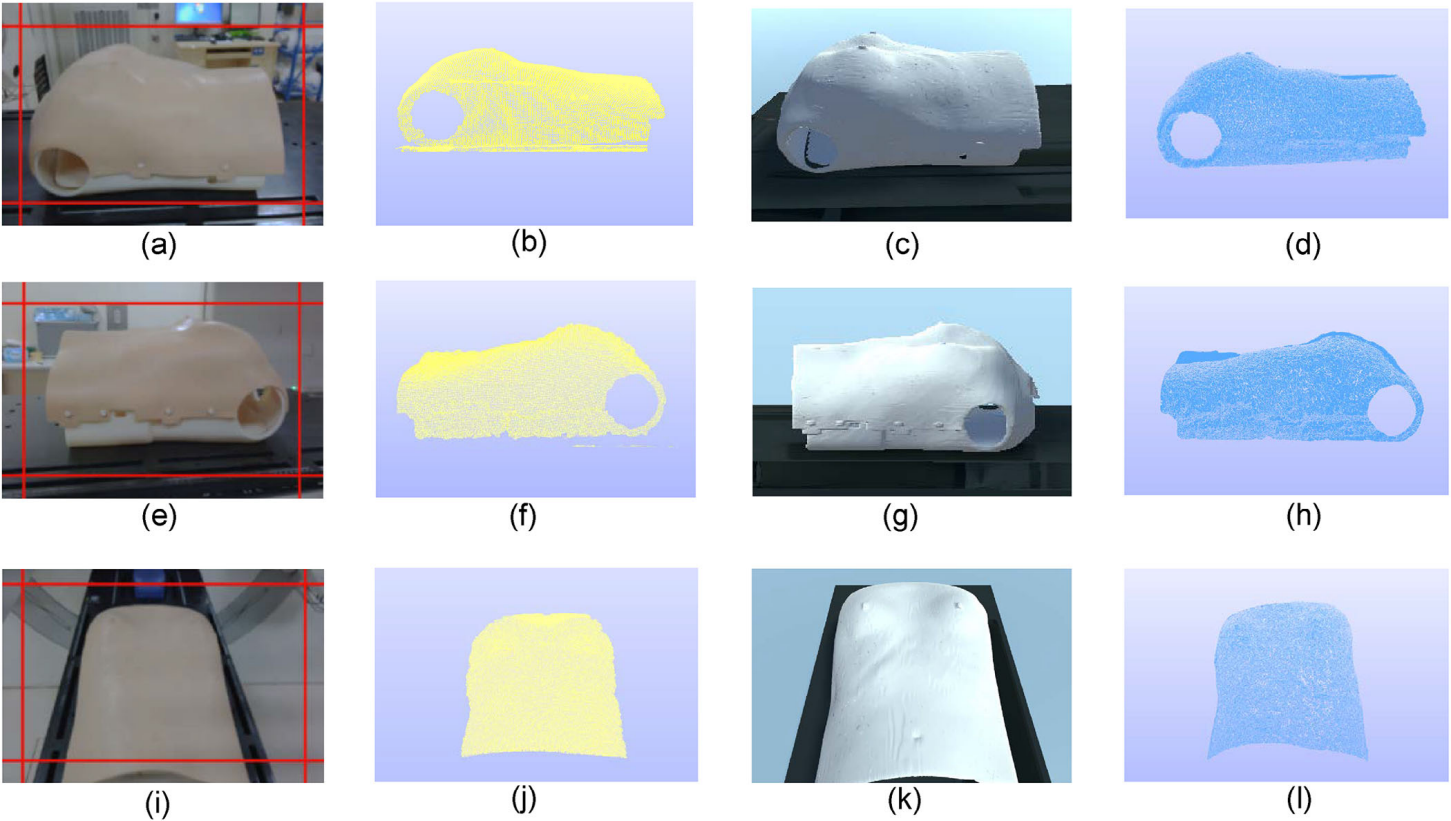
Visualization schematic of 3D imaging in the actual environment and 3D reconstruction in the simulation environment. The three perspectives are viewing the model from the left side, the right side, and from a top‐down perspective. The (a), (e), and (i) represent the actual captured images, with their corresponding surface imaging maps being (b), (f), and (j), respectively. The (c), (g), and (k) depict the simulated environment captured images, with their corresponding 3D reconstruction images being (d), (h), and (l) respectively.

To demonstrate the simulation capabilities of the system, 3D surface imaging of a human torso model was conducted in a radiotherapy room from three different angles in both real and simulated environments. From the left and right perspectives as well as from an aerial view, with an angle of 15 degrees with respect to the horizontal axis at a distance of 500 mm, an imaging of the surface was achieved under identical settings and 3D surface reconstruction algorithm. The CREALITY commercial 3D scanner CR‐Scan‐Ferret is fixed in place by a support structure in the radiation therapy room to scan the half‐body model from different angles for actual 3D reconstruction. The manufacturer states that the scanner can achieve an accuracy of 0.1 mm. Subsequently, a complete virtual model was reconstructed through CT scanning of the torso, which was then placed in the simulation system for 3D reconstruction to obtain virtual surface imaging results. The specific CT reconstruction method refers to a previous study.^13^ The results from the scanner and simulation were aligned using ICP registration, with RMSE values of 0.46, 0.47, and 0.52 mm calculated between the two point clouds, as shown in Figure 15. The figures illustrate that the simulation system effectively reconstructs 3D models and generates surface imaging point cloud data.

### 4.3 Verification in SGRT algorithm development

**FIGURE 16:**
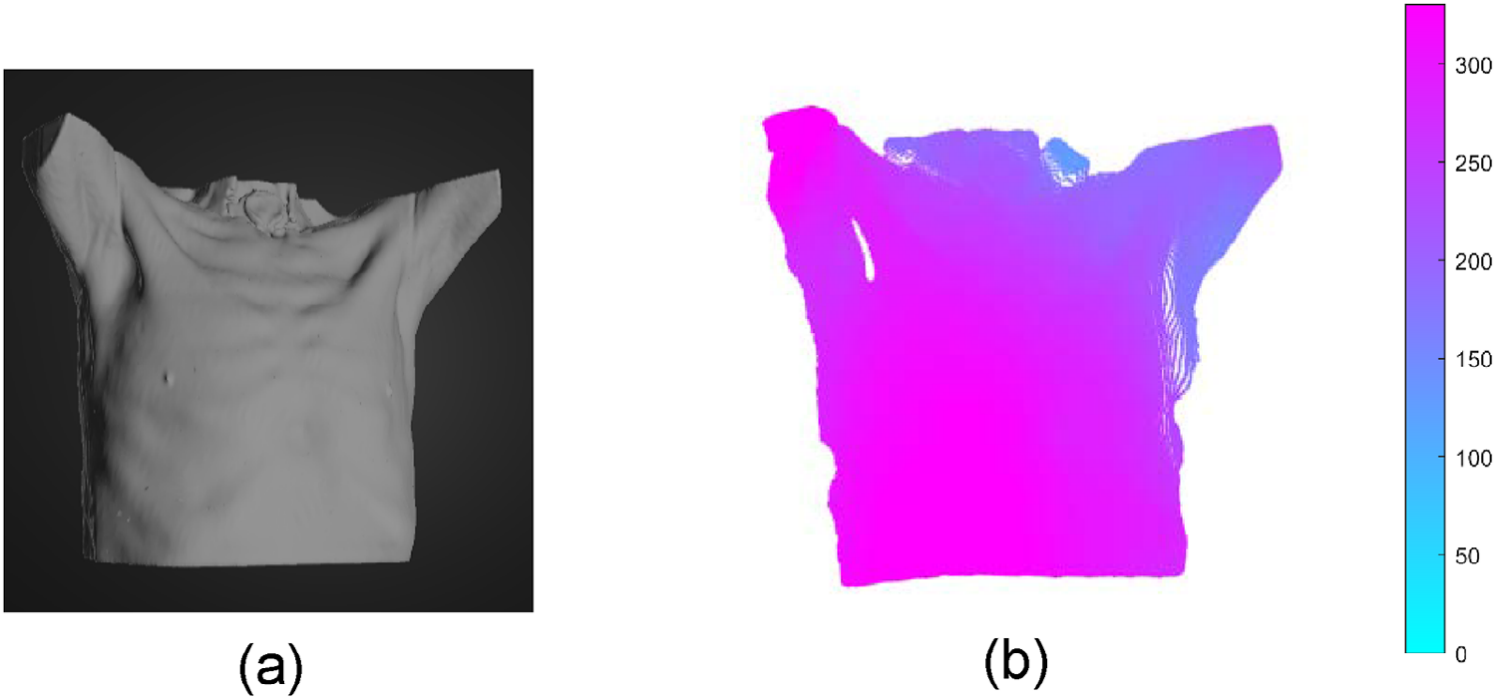
The schematic diagram of the skin surface reconstructed by the simulation system during tumor tracking. (a) The CT reconstruction model was placed in the simulation environment, (b) The skin surface reconstructed from the simulation system.

**FIGURE 17:**
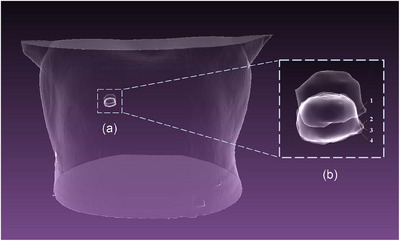
The tumor estimation results schematic diagram for patient 13. (b) The enlarged view of (a). In (b), 1 is the base model, 2 and 3 are the tumor estimation results of the EOI phase under the simulation system and simulated noise obtained by deforming the base model, and 4 represents the tumor surface at the EOI phase (ground truth). EOI, end‐of inhalation

We conducted algorithm validation of the surface imaging of the simulation system using the tumor tracking method from a previous study.^13^ This validation was performed on 4D‐lung datasets of 15 patients at the Léon Bérard Cancer Center. The skin surface reconstructed from CT scans was placed in Unity for surface imaging, as shown in Figure 16. Each patient's respiratory motion phases were divided into 10 temporal phases, with each phase requiring the reconstruction of the skin surface in the simulation system. We employed the 3D skin surface images obtained from the simulation system, along with the skin surfaces acquired without simulated noise and with simulated Gaussian noise, as inputs for the tumor tracking algorithm. The tumor estimation was completed as depicted in Figure 17. In Figures 2, 17, and 3 represent the tumor estimation results at the end of inhalation (EOI) under two different conditions: one using surface imaging data obtained from the simulation system and the other using surface imaging data from CT segmentation with simulated noise. Subsequently, the computed errors were compared to validate the applicability of the simulation system. It's important to note that both cases, without and with simulated noise, used the skin surface directly obtained from CT reconstruction segmentation, without utilizing the simulation system for surface imaging. Tables 4 and 5 show that the mean tumor position errors (TPE) for cases without simulated noise, with simulated Gaussian noise with a standard deviation of 0.5 mm, and simulated by the simulation system were 1.29, 1.33, and 1.57 mm, respectively. The corresponding mean dice similarity coefficient (DSC) values were 0.924, 0.920, and 0.906. It has effectively validated that the surface imaging achieved by the simulation system can be used for the development of tumor tracking algorithms. Based on the quantitative analysis of errors in the simulation system (with simulation errors below 1 mm), these results demonstrate that the simulation system, by simulating consistent imaging characteristics and error features of real scenarios with the real environment, can better reflect the true level of SGRT tumor tracking algorithms in clinical settings compared to using highly idealized surface images as inputs. This demonstrates the potential application value of the simulation system in the development of the SGRT algorithm, significantly enhancing the clinical relevance of algorithm validation.

**TABLE 4:** Mean ± standard TPE on each phase without simulated noise, and the validation results with simulated noise and using the simulation system. (unit: mm).

Patient no.	Without simulated noise (mm)	With simulated noise (mm)	Simulation system (mm)
**1**	1.22 ± 0.57	1.35 ± 0.61	1.53 ± 0.62
**2**	0.77 ± 0.29	0.84 ± 0.29	1.07 ± 0.33
**3**	1.26 ± 0.47	1.50 ± 0.49	1.70 ± 0.51
**4**	0.74 ± 0.70	0.79 ± 0.69	1.02 ± 0.68
**5**	0.78 ± 0.22	0.78 ± 0.22	1.01 ± 0.23
**6**	2.86 ± 1.41	2.76 ± 1.51	3.08 ± 1.55
**7**	1.05 ± 0.50	1.02 ± 0.56	1.27 ± 0.58
**8**	1.26 ± 0.52	1.19 ± 0.57	1.50 ± 0.49
**9**	1.55 ± 0.52	1.53 ± 0.52	1.79 ± 0.54
**10**	1.46 ± 0.93	1.53 ± 0.75	1.70 ± 0.81
**11**	2.20 ± 1.15	2.15 ± 0.93	2.42 ± 0.96
**12**	1.08 ± 0.24	1.08 ± 0.24	1.31 ± 0.26
**13**	1.44 ± 0.42	1.54 ± 0.29	1.73 ± 0.39
**14**	0.92 ± 0.29	0.96 ± 0.32	1.24 ± 0.33
**15**	0.80 ± 0.36	0.94 ± 0.22	1.19 ± 0.35
**Mean ± std**	1.29 ± 0.84	1.33 ± 0.83	1.57 ± 0.86

Abbreviation: TPE, tumor center position error.

**TABLE 5:** Mean ± standard DSC metric between real tumor surfaces and model‐generated tumor surfaces for 15 patients on each phase without simulated noise, and the validation results with simulated noise and using the simulation system.

Patient No.	Without simulated noise (mm)	With simulated noise (mm)	Simulation system (mm)
**1**	0.943 ± 0.008	0.940 ± 0.006	0.926 ± 0.012
**2**	0.972 ± 0.013	0.970 ± 0.011	0.958 ± 0.027
**3**	0.915 ± 0.015	0.908 ± 0.015	0.894 ± 0.040
**4**	0.965 ± 0.033	0.956 ± 0.024	0.941 ± 0.038
**5**	0.986 ± 0.005	0.986 ± 0.005	0.962 ± 0.015
**6**	0.828 ± 0.138	0.824 ± 0.020	0.813 ± 0.141
**7**	0.920 ± 0.015	0.916 ± 0.015	0.905 ± 0.022
**8**	0.891 ± 0.022	0.881 ± 0.042	0.870 ± 0.046
**9**	0.910 ± 0.114	0.910 ± 0.014	0.892 ± 0.130
**10**	0.896 ± 0.016	0.895 ± 0.013	0.878 ± 0.049
**11**	0.871 ± 0.028	0.869 ± 0.021	0.856 ± 0.033
**12**	0.981 ± 0.071	0.985 ± 0.004	0.969 ± 0.055
**13**	0.893 ± 0.031	0.878 ± 0.045	0.871 ± 0.039
**14**	0.941 ± 0.009	0.937 ± 0.013	0.923 ± 0.028
**15**	0.949 ± 0.014	0.948 ± 0.022	0.935 ± 0.031
**Mean ± std**	0.924 ± 0.043	0.920 ± 0.046	0.906 ± 0.052

Abbreviation: DSC, dice similarity coefficient.

## 5 DISCUSSION

The virtual 3D surface imaging system for validating SGRT tumor tracking algorithms was proposed. This system performed surface imaging of both regular models and human models in a virtual environment, and was validated in the lung tumor tracking algorithm. However, during the surface imaging process, imaging from different perspectives and distances may result in certain limitations on field reconstruction, leading to increased reconstruction errors. If specific clinical radiotherapy guidance relies on the defect area, accuracy loss may inevitably occur. This limitation can be partially addressed through multi‐position imaging, a consideration we will incorporate into our future research endeavors. Additionally, the validation of the simulation system is primarily based on standard human torso models. In the SGRT process, severe skin folds may cause excessive distortion of structural light patterns, leading to inaccuracies in the simulation. In such cases, customized adjustments based on individual anatomical differences are essential for simulating complex cases, such as those involving obese patients, pediatric patients, or post‐operative scar patients. Future work can address this issue through continuous optimization of the Unity physics engine. The simulation system can offer customization features, supporting the creation of individualized models and generating a database tailored for clinical applications. In addition to its application in the development of SGRT algorithms, the proposed simulation system has the potential to be used in generating machine learning datasets. By simulating a space identical to the experimental environment, the cost of generating datasets can be significantly reduced, and they can be used for validating various 3D reconstruction algorithms.

## 6 CONCLUSION

In this study, a simulation for structured light 3D reconstruction was completed using the Unity platform and employing PMP. The 3D reconstruction results obtained from simulation can be utilized as surface imaging data for the development of the SGRT tumor tracking algorithms. The RMSE of the surface imaging point clouds between the simulation system and the actual environment from different perspectives was all less than 1 mm. This demonstrates the strong modeling capabilities of the simulation system. Factors affecting reconstruction accuracy, such as the relative position between the camera and the surface of the object under test and ambient light intensity, were verified. The simulation system demonstrates high precision and reliability and can also be utilized to assist in the validation of the SGRT tumor tracking algorithms.

## AUTHOR CONTRIBUTIONS

All authors contributed to the study conception and design. Xiaolong Wu and Junchao Qian analyzed and interpreted data for the work. The first draft of the manuscript was written by Xiaolong Wu. Final approval of the work to be published was finished by Junchao Qian.

## CONFLICT OF INTEREST STATEMENT

The authors declare no conflicts of interest.
